# In Situ Measurement of the Strain Field at the Fatigue Crack Tip Based on Sub-Image Stitching and Matching DIC

**DOI:** 10.3390/ma15155150

**Published:** 2022-07-25

**Authors:** Zhiyuan Lin, Hongbin Shang, Hongli Gao, Xinwei Huang

**Affiliations:** Key Laboratory of Special Equipment Manufacturing, Advanced Processing Technology of the Ministry of Education, Zhejiang University of Technology, Hangzhou 310023, China; 2112002035@zjut.edu.cn (Z.L.); s534314450@163.com (H.S.); huang_xin_wei@163.com (X.H.)

**Keywords:** in situ fatigue crack propagation, micro DIC, image stitching, template matching, strain field

## Abstract

Studying the in situ measurement and evolution of the strain field at the crack tip during fatigue crack growth (FCG) is of great significance for understanding the fracture characteristics of materials and predicting fatigue life. Herein, a new method is proposed for the in-situ measurement of the strain field at the fatigue crack tip based on microscopic digital image correlation (DIC). The method proposed solves the problem of the existing in situ strain field measurement method being unable to dynamically track the crack tip and take the crack tip image due to the limitation of the field of view of the microscopic camera. A macroscopic camera is used to capture the global crack images on one side of the compact tension (CT) specimen. Meanwhile, a microscopic camera is used to track and capture the crack propagation speckle image on the other side of the CT specimen. The proposed method was verified by experiments with Quenching and Partitioning 980 (Q&P980) steel, and the results showed that the method has high accuracy, with the average measurement error being less than 5% and the maximum error being less than 10%. A butterfly shape of the measured strain field and the strain concentration near the crack tip were observed. The success of this method will help to obtain better insight into and understanding of the fracture behavior of metal materials as well as precise prediction of the fatigue life of metal materials.

## 1. Introduction

Fatigue failure is the main form of failure of metal mechanical parts. When the metal material is subjected to alternating loads, the initiation and propagation of fatigue cracks occur, which may lead to the fatigue failure behavior of the material. Based on existing studies, the fracture behavior of metal parts is closely related to the initiation and propagation of fatigue cracks [1,2,3]. As early as the 1960s, a method of studying the fatigue crack propagation mechanism based on strain measure and analysis was proposed [4,5]. In the past ten years, there have been many achievements in studying the fatigue crack propagation mechanism by studying the evolution law of the strain field at the crack tip. Alshammrei et al. [6] combined DIC and finite element analysis to conduct full-field mechanical characterization of fatigue crack growth of 316L stainless steel under cyclic loading conditions. The evolution of the strain field at the crack tip was observed, and the effect of crack closure behavior on crack growth was evaluated. Malitckii et al. [7] used DIC technology to study the strain accumulation during the propagation of microstructural small fatigue cracks in 18%Cr ferritic stainless steel polycrystalline materials. Kujawski [8] proposed a simple analysis method to estimate the elastic–plastic strain and stress in front of the stable growth fatigue crack tip, which was in good agreement with the DIC calculation results.

DIC is a non-interference and non-contact precise optical measurement method based on digital image processing, which is used to measure the global displacement and strain field of the loaded body [9,10,11]. For measurement of the deformation field in the crack tip region, DIC is better than other global photometric methods, including holographic interferometry [12], Moiré interferometry [13,14], and laser speckle photography technology [15,16]. It has the characteristics of higher accuracy, stronger applicability, and multiscale measurement ability. In addition, DIC has lower requirements for experimental conditions, and only needs to make speckle images on the surface of the specimen to perform in- and ex-situ measurements at different lengths and scales. Ex-situ DIC can provide higher resolution strain field measurements [17,18,19], but the strain field can only be measured on unloaded specimens, and the measured data are residual strain data. In-situ DIC is a better choice for observing the continuous distribution of the strain and displacement fields during crack propagation. A combination of the DIC technique and a microscopic camera system can provide enough spatial resolution to spatially and temporally measure the crack tip strain field during fatigue crack growth process. These characteristics have made micro-DIC the first choice for fatigue crack growth research in recent years, rather than scanning electron microscopy–digital image correlation (SEM-DIC) or synchrotron radiation X-ray devices that require more complex technology and specialized equipment [20]. Zhu et al. [20] studied the normal strain near the fatigue propagation crack of a 316L stainless steel compact tension (CT) specimen under the action of tensile cyclic load by micro-DIC. Based on the analysis of the displacement and strain measurement error, a method used to improve the precision of micro-DIC measurement was proposed. Lu et al. [21] used the in situ DIC technique to study the crack tip strain field of static and propagating fatigue cracks in CT specimens of 316L stainless steel under tensile-tensile cyclic loading. Wei Zhang et al. [22] performed high-resolution in-situ experiments on thin-plate specimens under cyclic loading with stepwise loading. The acquired microscopic images were analyzed by DIC to obtain the strain distribution at each loading step, and the change in the size of the plastic zone was further analyzed.

The main problem of existing in situ deformation field measurements carried out by micro-DIC is that the entire process of crack propagation cannot be continuously photographed due to the small field of view of the microscopic camera. At present, the solution is to adjust the microscopic camera to the crack tip position to collect the reference image and then apply the cyclic load to the specimen to perform the fatigue crack growth test. The target images of cracked specimens at different positions in a load cycle in which the fatigue crack propagates in the microscopic field of view are collected, and then the micro-strain field near the crack tip can be obtained. When the crack propagation is beyond the field of view of the microscopic camera, it is necessary to stop the fatigue crack growth test, adjust the camera to the new crack tip position to collect the reference image under the unloading condition, and then perform the fatigue crack growth test again to generate a new propagated crack. In this way, the propagation process of the crack, especially the long crack beyond the microscopic field of view, cannot be measured in situ, and crack tip deformation field data of the whole process of crack propagation cannot be obtained.

Therefore, a method for the in-situ measurement of the strain field at the crack tip based on sub-reference image stitching and matching combined with the micro-DIC technique is proposed in this paper. The sub-reference image stitching and matching algorithm include an image stitching algorithm based on scale invariant feature transform (SIFI) key point detection [23] and the progressive sampling consensus (PROSAC) fitting algorithm [24], and the image matching algorithm is based on a template matching algorithm. The image stitching algorithm is used to obtain the full-field reference image, and the template matching algorithm is used to find the sub-reference image corresponding to the target image of the crack tip in the whole crack propagation process in the full-field reference image. Through DIC calculation between the sub-reference image and the crack tip target image, the strain field of the growing fatigue crack tip can be obtained continuously. Among the various advanced steel grades, a third generation AHSS was proposed by Speer et al. in 2003, named quenching and partitioning steel (Q&P steel) [25]. Q&P steel with a final microstructure of martensite, retained austenite and/or ferrite, exhibits a combination of high strength and ductility, and has been considered as a potential candidate for automotive steel. The “980” in Q&P980 represents the grade of yield strength. Through low cycle fatigue crack growth testing of the CT specimen with a mode I notch made of the third generation ASHH steel Q&P980, fatigue cracks were generated and propagated continuously, in situ growing crack tip strain fields were measured using the proposed method, strain field data were analyzed, and the measurement accuracy was verified.

## 2. In Situ Measurement System of the Strain Field at the Fatigue Crack Tip

### 2.1. Overall Measurement Method

**Figure 1:**
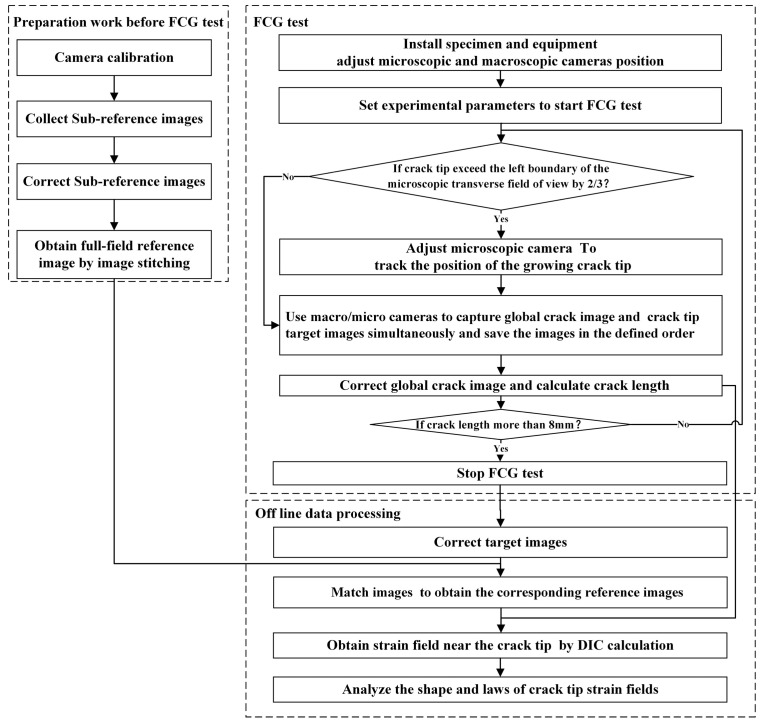
A flow chart of the in situ fatigue crack tip strain field measurement method.

A flow chart of the measurement method proposed in this paper is shown in Figure 1, which includes three stages: Preparation before the FCG test, the FCG test, and offline data processing. The macroscopic and microscopic cameras are calibrated with Zhang’s calibration method before the FCG test [26]. Two calibration boards with different specifications were made according to the field of view. Microscopic and macroscopic cameras take images from nine different angles separately. The distortion coefficients of the cameras and lens are calculated using the spatial locations of these images. The distortion coefficients of the cameras are used to correct the reference and target images. The corrected sub-reference images are stitched to obtain a full-field reference image. During the fatigue crack growth test, the microscopic camera tracks and captures the microscopic speckle image of the crack tip, while the macroscopic camera captures the global image of the macro crack. The speckle images are corrected to obtain target images, and the macroscopic global crack images are corrected to be used for calculating the crack length. The target images are used to search the full-field reference image through template matching so as to obtain the sub-reference images corresponding to the target images. The strain field data at the crack tip are calculated using the micro-DIC technique. The micro-DIC algorithm, image stitching algorithm, and template matching algorithm proposed in this paper are introduced in detail in Section 2.

### 2.2. Materials and Specimen

**Figure 2:**
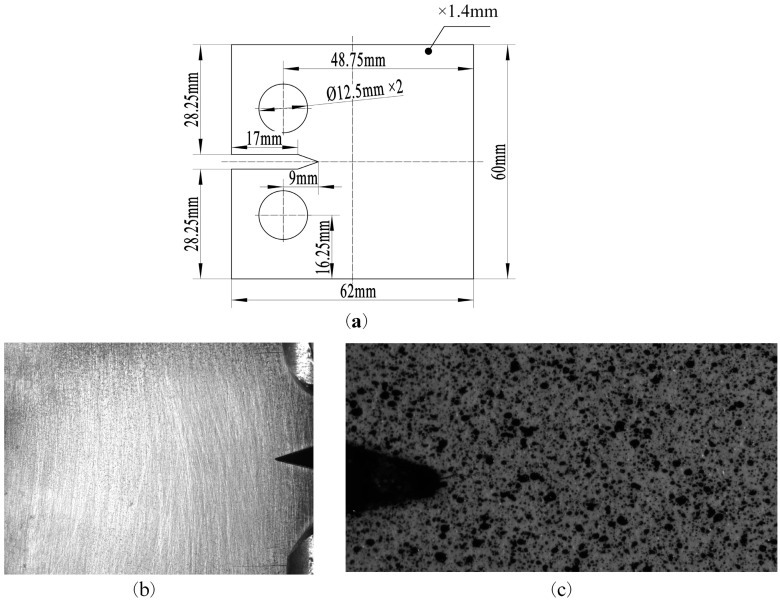
Parameters of the specimen’s geometry: (**a**) CT specimen size (thickness, 1.4 mm); (**b**) global crack image of the CT specimen; (**c**) digital speckle image of the CT specimen.

A test of the CT specimen was made of Q&P980 material with a mode I crack notch. The parameters of the specimen’s geometry are shown in Figure 2. One side of the specimen was polished into a smooth surface with a diffuse effect to show the crack path information, and a professional speckle spray pen was used to produce uniform microscopic speckles on the surface of the other side. A uniaxial tensile test of a dog bone specimen made of Q&P980 was carried out and the stress–strain curve was fitted by the Lamberg-Osgood (R-O) model [27]. The mechanical property parameters of Q&P980 were obtained by modifying the curve fitting results, as shown in Table 1.

The fitting process of the R-O curve is as follows: The stress–strain equation of the R-O model is shown in Equation (1).

(1) ε=εe+εp=σE+(σH)1n

where $\varepsilon_{e}$ and $\varepsilon_{p}$ are the elastic and plastic strains, respectively, ε is the total strain, and n is the hardening index. The R-O model is fitted using real stress–strain data. The formula for calculating the real stress and strain is shown in Equation (2):

(2) $$\tilde{\varepsilon }=\ln\frac{L_{i}+\Delta L}{L_{i}}=\ln\left(1+\frac{\Delta L}{L_{i}}\right)=\ln\left(1+\varepsilon \right)$$

(3) $$\tilde{\sigma }=\sigma \left(1+\varepsilon \right)$$

where $\tilde{\varepsilon }$ and $\tilde{\sigma }$ are the real strain and real stress.

**Figure 3:**
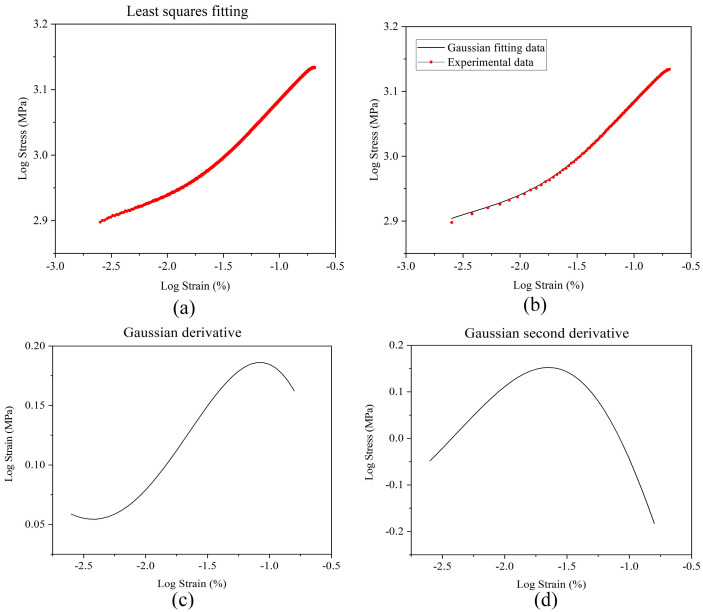
Fitting curve and extreme value analysis: (**a**) Least square fitting; (**b**) Gaussian fitting; (**c**) Gaussian derivative; (**d**) Gaussian second derivative.

The fitting results of the least square method are shown in Figure 3a. It can be found that the linearity of the fitting data is insufficient. This shows that the plastic strain formula of Q&P980 cannot fully characterize the plastic segment. It is noted that the fitted curve can be separated into two straight lines, thus considering the classical logarithm fitting function (second-order Gaussian fitting). The second-order Gaussian function is shown in Equation (4), and the Gaussian fitting curve is shown in Figure 3b. The first and second derivatives of the Gaussian curve are shown in Figure 3c,d. The extreme point of the second derivative curve is solved as the boundary point of the piecewise straight line. The result is −1.65, with a converted strain of 0.022. The stress corresponding to this strain value is 918.80 MPa.

(4) $$G\left(x\right)=a_{1}e^{-{\left(\left(x-b_{1}\right)/c_{1}\right)}^{2}}+a_{2}e^{-{\left(\left(x-b_{2}\right)/c_{2}\right)}^{2}}$$

**Figure 4:**
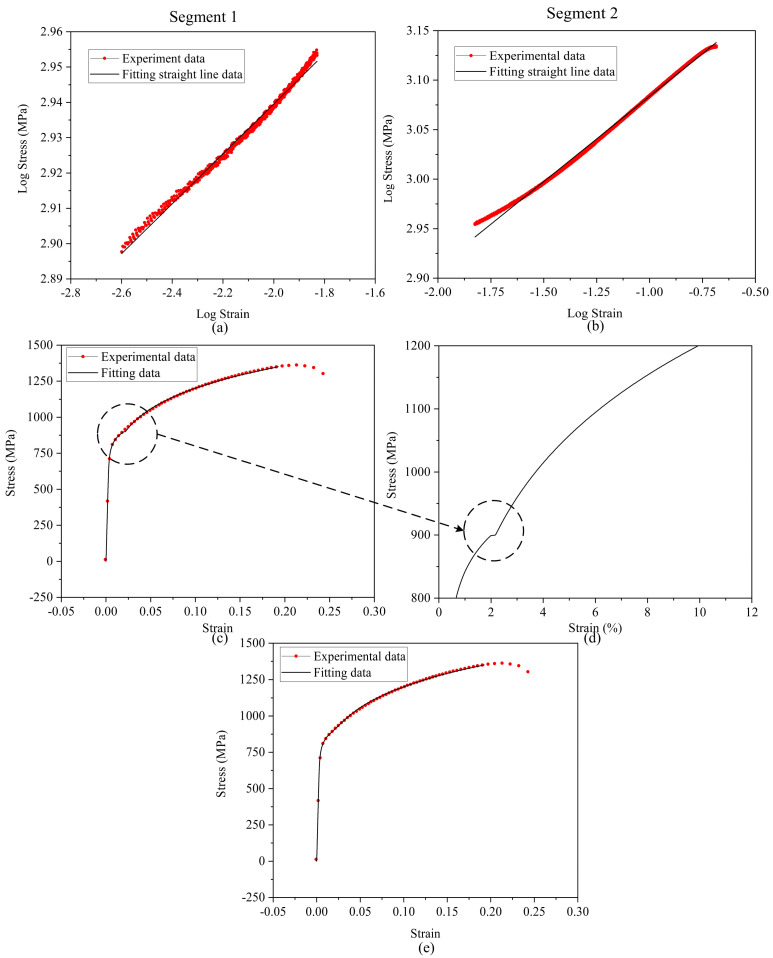
Fitting results: (**a**) Segment 1 fitting results; (**b**) segment 2 fitting results; (**c**) fitting results; (**d**) region near the intersection of two curves; (**e**) final fitting results.

Based on the segmented data, Gaussian fitting was continued. The fitting results are shown in Figure 4a,b. The results show that piecewise fitting has better linearity. The R-O constitutive equation was modified, and the modified results are shown in Equation (5):

(5) $$\{\begin{array}{c} \varepsilon =\frac{\sigma }{197200}+{\left(\frac{\sigma }{1205.03594}\right)}^{1/0.07072}\;\;\;\;\;\sigma <918.80\mathrm{MPa}\\ \varepsilon =\frac{\sigma }{197200}+{\left(\frac{\sigma }{1803.017741}\right)}^{1/0.1723}\;\;\;\;\;\sigma \geq 918.80\mathrm{MPa} \end{array}$$

The fitting results compared to the original data are shown in Figure 4c. The overall fitting is good. However, as shown in Figure 4d, the results near the intersection of two curves are poor. The common tangent line of two curves was calculated as the transition curve. The calculation method of the common tangent line is digital analog iterative calculation. The final R-O fitting results are shown in Figure 4e, and the modified R-O constitutive equation is shown in Equation (6). The mechanical property parameters were obtained as shown in Table 1.

(6) $$\{\begin{array}{c} \varepsilon =\frac{\sigma }{197200}+{\left(\frac{\sigma }{1205.03594}\right)}^{\frac{1}{0.07072}}\;\;\;\;\;\;\sigma <874.47\mathrm{MPa}\\ \varepsilon =1.7814\times 10^{-4}\sigma -0.1412\;\;\;874.47\leq \sigma <980.22\mathrm{MPa}\\ \varepsilon =\frac{\sigma }{197200}+{\left(\frac{\sigma }{1803.017741}\right)}^{\frac{1}{0.1723}}\;\;\;\;\;\sigma \geq 980.22\mathrm{MPa} \end{array}$$

Speckle quality directly affects the accuracy of DIC calculation. In this paper, the average gray gradient and the average gray second derivative were used to evaluate the speckle quality. The average gray gradient is the main index, and the calculation formula is shown in Equation (7):

(7) $$\delta_{f}=\frac{\sum_{i=1}^{W}\sum_{j=1}^{H}\sqrt{f_{x}{\left(x_{ij}\right)}^{2}+f_{y}{\left(y_{ij}\right)}^{2}}}{W*H}$$

where *W* and *H* are the width and height of the image (in pixels), respectively; $f_{x}{\left(x_{ij}\right)}^{2}$ and $f_{y}{\left(y_{ij}\right)}^{2}$ are the grayscale derivatives of pixel $x_{ij}$ in the  x  and y directions, respectively, which can be calculated using commonly used gradient operators.

The second derivative of the average gray level was used as an auxiliary evaluation index, as shown in Equation (8):

(8) $$\omega_{f}=\frac{\sum_{i=1}^{W}\sum_{j=1}^{H}\sqrt{f_{xx}{\left(x_{ij}\right)}^{2}+f_{yy}{\left(y_{ij}\right)}^{2}}}{W*H}$$

In practice, the average gray gradient is preferred for calculation, and the larger the $\delta_{f}$, the better the speckle quality. When $\delta_{f}$ is the same, the second derivative of the average gray level is further calculated, and the smaller the $\omega_{f}$, the better the speckle quality.

### 2.3. Measurement System Components

**Figure 5:**
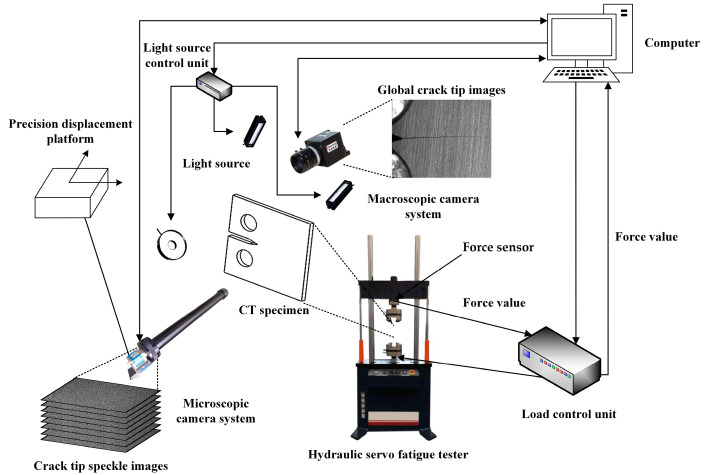
In situ measurement system components.

**Figure 6:**
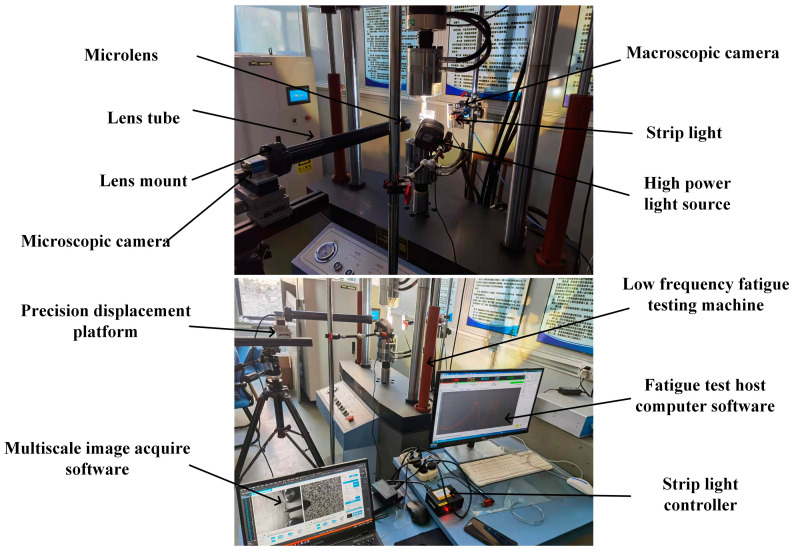
Experimental platform.

The composition of the measurement system is shown in Figure 5, which mainly includes a low cycle fatigue test system and a multiscale fatigue crack image acquisition system. The experimental set up is shown in Figure 6. The low cycle fatigue test system is composed of a hydraulic servo fatigue tester, specimen, force sensor, load control unit, and precision displacement platform. The hydraulic servo fatigue tester is PWS-50, produced by Tianshui Hongshan Company, with a maximum load of 50 kN. The multiscale fatigue crack image acquisition system includes a computer with synchronous image acquisition software, the proposed digital image processing software, VIC-2D software, a macroscopic camera system, a microscopic camera system, and a camera light source. The synchronous image acquisition software controls the fixed macroscopic camera and tracking microscopic camera to capture images at the same time on both sides of the CT specimen by receiving the force signal feedback from the load control unit. 

**Figure 7:**
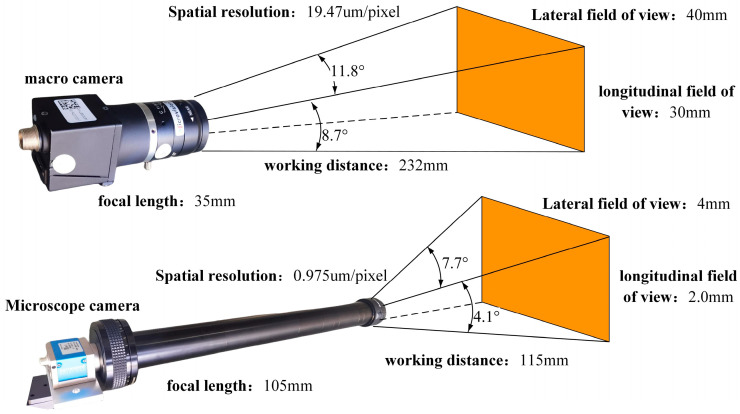
Camera parameters.

The parameters of the macro and micro acquisition systems are shown in Figure 7. The macroscopic focal length of the camera is 35 mm, the working distance is 232 mm, the field of view under this working distance is 40 × 30 mm, and the corrected spatial resolution is 19.47 μm/pixel. The microscopic camera uses a telephoto lens set. In this paper, the maximum magnification was used to shoot the minimum field of view. The focal length of the camera is 105 mm, the working distance is 115 mm, the field of view is 4 × 2.11 mm, and the spatial resolution of the corrected image is 0.97 μm/pixel.

### 2.4. Multiscale Fatigue Crack and Speckle Image Acquisition

The in-situ image acquisition operation is controlled through the self-developed multiscale image acquisition software, as shown in Figure 6, which includes the functions of tracking load value acquisition, crack length detection, and speckle quality evaluation. For a short crack whose length is less than 2 mm, the crack length cannot be accurately measured by the image captured by the macroscopic camera. In this paper, according to the method of reference [28], virtual extensometer technology was used to realize accurate measurement of the short crack length.

**Figure 8:**
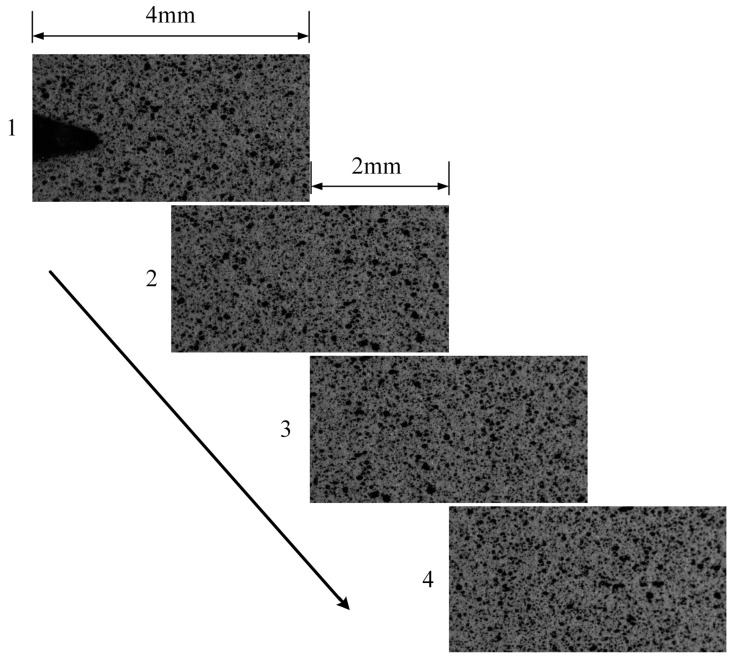
Sub-reference images.

The speckle image acquisition operation consists of two steps, namely the acquisition of reference images before the fatigue propagation test and acquisition of crack tip target images during fatigue crack growth. The maximum fatigue crack length of this test was set to approximately 8 mm, and the microscopic camera moving step length was set to 2 mm according to the size of the microscopic transverse field of view of 4 mm. Four speckle sub-reference images were collected before the FCG test by moving the microscopic camera three times along the crack propagation direction with a 2 mm step length, as shown in Figure 8. Movement of the microscopic camera was accomplished through the precision displacement platform, as shown in Figure 6. After the speckle image had been captured, the microscopic camera was returned to the position where the first image was captured.

The load parameters of the FCG test are as follows: Average load value of 1.8 kN, amplitude value of 1.2 kN, fatigue testing frequency of 8 Hz. According to the previous test experience, the fatigue crack can grow to approximately 15 mm stably under these test conditions. In the process of the fatigue crack growth test, the position of the microscopic camera was adjusted by observing the approximate position of the crack tip in the microscopic field of view. The position of the microscopic camera was adjusted to ensure that the collected crack tip was not at the edge of the image. Seven groups of images with cycle numbers of 5300, 10,000, 15,000, 20,000, 22,500, 25,000, and 27,000 were collected. When collecting images, the vibration frequency of the fatigue tester needs to be reduced to 0.01 Hz. The multiscale image acquisition software receives the force signal from the load control unit, and when the force value is equal to the set acquisition load value, it sends an acquisition signal to the microscopic camera. The microscopic camera collects one picture every time it receives an acquisition signal. Meanwhile, the related force values should be recorded. When the maximum load is reached, the macroscopic camera is triggered to capture a global crack image. Finally, each group with 26 microscopic speckle images and one macroscopic global crack image can be obtained and saved on the computer in a defined order.

**Figure 9:**
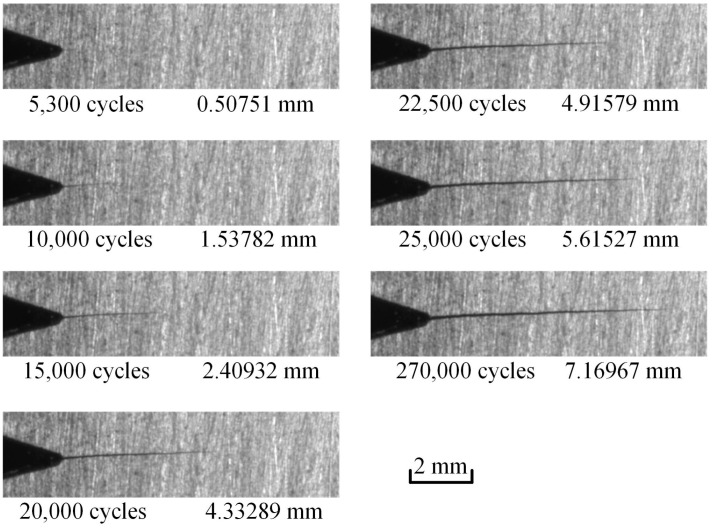
Global crack images corresponding to different cycle numbers.

**Figure 10:**
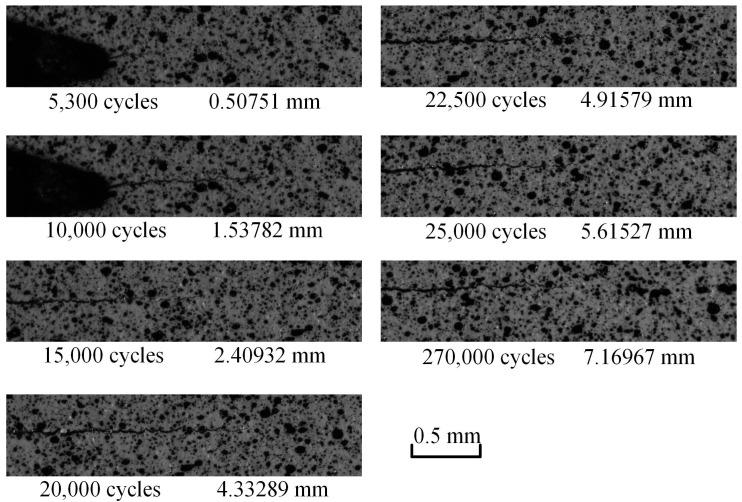
Speckle images corresponding to different cycle numbers.

Seven speckle images and seven macroscopic global crack images at the maximum load corresponding to different cycle numbers are shown in Figure 9 and Figure 10, respectively.

## 3. In Situ Measurement Algorithms of the Strain Field at the Fatigue Crack Tip

### 3.1. Strain Field Measurement Algorithm of the Fatigue Crack Tip Based on DIC

**Figure 11:**
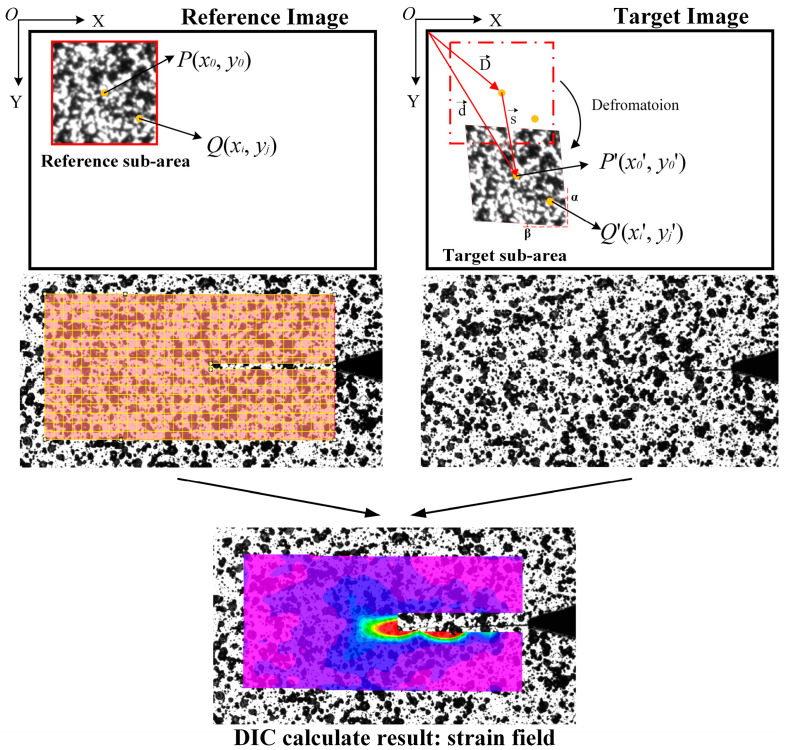
DIC Principle.

The principle of the DIC algorithm is shown in Figure 11 [9]. A reference image of the specimen before deformation is collected and divided into several sub-regions. $P\left(x_{0},y_{0}\right)$ is the center of the reference sub-area in the reference image, and $P^{\prime }\left(x_{0}^{\prime },y_{0}^{\prime }\right)$ is the center of the target sub-area matched by the correlation function in the target image. The target sub-area is the result of the deformation of the reference sub-area. $Q^{\prime }\left(x_{i}^{\prime },y_{i}^{\prime }\right)$ in the target sub-area is the point corresponding to another point $Q\left(x_{i},y_{i}\right)$ in the reference sub-area. The displacement of $Q^{\prime }\left(x_{i}^{\prime },y_{i}^{\prime }\right)$ relative to $Q\left(x_{i},y_{i}\right)$ is calculated by the I-order shape function.

(9) $$x_{i}^{\prime }=x_{i}+\xi_{1}\left(x_{i},\;y_{i}\right)$$

(10) $$y_{i}^{\prime }=y_{i}+\eta_{1}\left(x_{i},y_{i}\right)$$

The expansion of the first-order shape function $\xi_{1}\left(x_{i},\;y_{i}\right)$, $\eta_{1}\left(x_{i},y_{i}\right)$ is as follows:

(11) $$\xi_{1}\left(x_{i},y_{i}\right)=\mu +\mu_{x}\Delta x+\mu_{y}\Delta y$$

(12) $$\eta_{1}\left(x_{i},y_{i}\right)=\nu +\nu_{x}\Delta x+\nu_{y}\Delta y$$

where *μ* represents the displacement of the center of the reference sub-region in the x-axis direction $\mu =x_{0}^{\prime }-x_{0}$; ν  indicates the displacement of the reference sub-center in the y-axis direction; $\mu_{x}$, $\mu_{y}$, $\nu_{x}$, and $\nu_{y}$ are the partial derivatives of the central displacement of the reference sub-region in the x- and y-axis directions, indicating the displacement gradient of the reference sub-region; $\Delta x=x_{i}-x_{0}$ and $\Delta y=y_{i}-y_{0}$ denote the distance from point $Q\left(x_{i},y_{i}\right)\;$ to the center $P\left(x_{0},y_{0}\right)$ of the reference sub-area. As a result, the displacement of each point in each sub-region of the target image relative to the reference image can be obtained.

For example, in Figure 11, the definition of the deformation gradient is as follows:

(13) $$F=\frac{\partial d}{\partial D}=I+\frac{\partial s}{\partial D}$$

where *D* is the vector (O, P), *d* is the vector (O, P′), *s* is the vector (P, P′), and *I* is the unit tensor. *F* is expanded into matrix form:

(14) $$F=\left[\begin{array}{cc} \frac{\partial x}{\partial X} & \frac{\partial x}{\partial Y}\\ \frac{\partial y}{\partial X} & \frac{\partial y}{\partial Y} \end{array}\right]=\left[\begin{array}{cc} 1+\frac{\partial u}{\partial X} & \frac{\partial u}{\partial Y}\\ \frac{\partial v}{\partial X} & 1+\frac{\partial v}{\partial Y} \end{array}\right]$$

where (X, Y) refers to the pre-deformation coordinates; (x, y) refers to the coordinates after deformation; u=x−X=tanα·Y; v=y−Y=tanβ·X.

**Figure 12:**
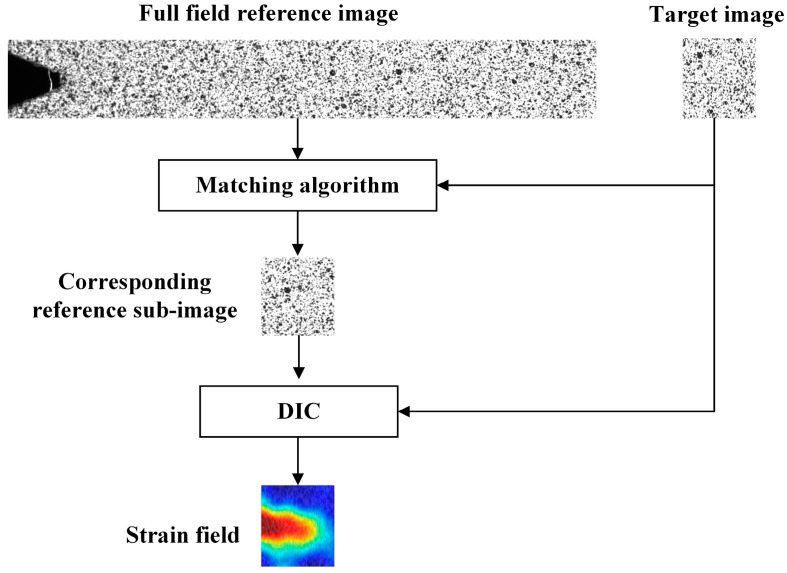
DIC full-field strain calculation of the crack tip region.

According to Equation (14), the deformation gradient of Figure 12 is as follows:

(15) $$F=\left[\begin{array}{cc} 1 & \tan\alpha \\ \tan\beta & 1 \end{array}\right]$$

The calculated Lagrange strain tensor is as follows:

(16) $$E=\frac{1}{2}\left(\left[\begin{array}{cc} 1+\tan^{2}\beta & \tan\alpha +\tan\beta \\ \tan\alpha +\tan\beta & 1+\tan^{2}\alpha \end{array}\right]-\left[\begin{array}{cc} 1 & 0\\ 0 & 1 \end{array}\right]\right)\;\;\;=\frac{1}{2}\left[\begin{array}{cc} \tan^{2}\beta & \tan\alpha +\tan\beta \\ \tan\alpha +\tan\beta & \tan^{2}\alpha \end{array}\right]$$

The strain components obtained from the Lagrange strain tensor E are as follows:

(17) $$e_{xx}=\frac{1}{2}\tan^{2}\beta ,\;\;e_{yy}=\frac{1}{2}\tan^{2}\alpha ,\;\;e_{xy}=\frac{1}{2}\left(\tan\alpha +\tan\beta \right),\;\;e_{zz}=e_{xz}=e_{yz}=0$$

According to the von Mises yield criterion, the von Mises equivalent strain field is calculated using Equation (18):

(18) $$\bar{e}=\frac{\sqrt{2}}{3}\cdot \sqrt{A^{2}+B^{2}+C^{2}+6D}$$

where $A=e_{xx}-e_{yy};B=e_{yy}-e_{zz};A=e_{xx}-e_{zz};D=e_{xy}{}^{2}-e_{xz}{}^{2}+e_{yz}{}^{2}$.

The key of the in-situ measurement algorithm for the crack tip strain field proposed in this paper is to obtain the reference sub-image matching with the target image. The principle is shown in Figure 12. The corresponding reference sub-image is obtained by template matching the target image with the full-field reference image, and then calculated using the DIC algorithm to obtain the crack tip strain field.

### 3.2. Reference Sub-Image Stitching Algorithm

**Figure 13:**
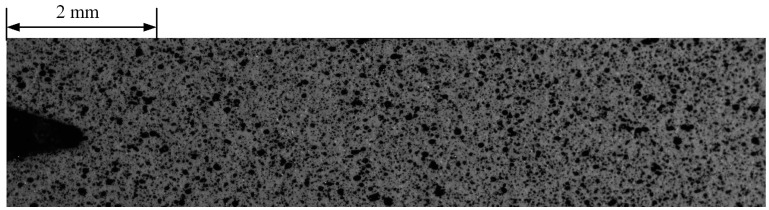
Full-field reference image.

The technology of image stitching is actually used to find the same feature points of two images and to establish the mapping relationship between the two groups of feature points. One image is calculated through the mapping relationship and superimposed on the other image to complete the image stitching. Considering that speckle images have the high robustness of SIFI key point distribution, the feature points of the two images to be stitched are obtained by detecting the SIFI key points, and the effective feature points are screened out. The relationship between the feature points of the two images to be stitched is obtained by PROSAC fitting algorithm, which is represented by the form of a homography matrix. As shown in Figure 8, four sub-reference images were obtained. The full-field reference image obtained using the image stitching algorithm according to the order in Figure 8 is shown in Figure 13. The calculated homography matrix is shown in Table 2.

It was found that the translation of the main x-direction of the homography matrix is mainly in the x-direction and the displacement is approximately 2501 pixels. It is confirmed that the moving distance is close to half of the microscopic field of view. The coefficients of translation or rotation in other directions were negligible. On the whole, the element composition of the transformation matrix is in line with expectations. The accuracy of the splicing results was proven from the side.

### 3.3. Corresponding Reference Sub-Image Template Matching Algorithm

Image matching uses a template matching algorithm to search the sub-reference image corresponding to the target image in the full-field reference image. The matching of the reference and target images can be guaranteed using the correlation functions shown in Equation (19), which can eliminate the influence of image brightness.

(19) $$C_{ZNSSD}=\overset{M}{\underset{i=-M}{\sum }}\overset{M}{\underset{j=-M}{\sum }}{\left[\frac{f\left(x_{i},y_{i}\right)-f_{m}}{\sqrt{\sum_{i=-M}^{M}\sum_{j=-M}^{M}{\left[f\left(x_{i},y_{i}\right)-f_{m}\right]}^{2}}}-\frac{g\left(x_{i}^{\prime },y_{j}^{\prime }\right)-g_{m}}{\sqrt{\sum_{i=-M}^{M}\sum_{j=-M}^{M}{\left[g\left(x_{i}^{\prime },y_{j}^{\prime }\right)-g_{m}\right]}^{2}}}\right]}^{2}$$

where:

(20) $$\{\begin{array}{c} f_{m}=\frac{1}{{\left(2M+1\right)}^{2}}\overset{M}{\underset{i=-M}{\sum }}\overset{M}{\underset{j=-M}{\sum }}f\left(x_{i},y_{i}\right)\\ g_{m}=\frac{1}{{\left(2M+1\right)}^{2}}\overset{M}{\underset{i=-M}{\sum }}\overset{M}{\underset{j=-M}{\sum }}g\left(x_{i}^{\prime },y_{j}^{\prime }\right) \end{array}$$

where $f_{m}\;g_{m}$ represents the gray average values of the reference and target sub-areas, respectively; $f\left(x_{i},y_{i}\right)$ is the pixel gray value of the reference sub-region with the coordinate of $\left(x_{i},y_{i}\right)$; $g\left(x_{i}^{\prime },y_{j}^{\prime }\right)$ is the pixel gray value with the coordinates of $\left(x_{i}^{\prime },y_{j}^{\prime }\right)$ in the target sub-area. The sub-area with the greatest correlation is the reference sub-area corresponding to the target image. The sub-reference image corresponding to the target image of any crack length can be obtained using the method of template matching.

## 4. Results and Discussion

### 4.1. Verification of Image Stitching and the Template Matching Algorithm

**Figure 14:**
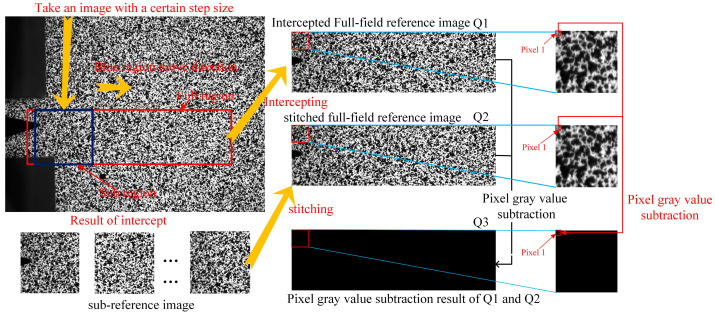
Verification process of image stitching.

In the FCG test, a microscopic camera was used, which had a small field of view and was not suitable for verifying the correctness of the image stitching algorithm proposed in this paper. We used a group of additional macroscopic speckle images to illustrate the verification process, as shown in Figure 14. Q1 is a simulated full-field reference image intercepted from a red rectangle in an entire speckle image. Q2 is the stitched full-field reference image of a group of sub-images intercepted by a blue rectangular region in a red rectangular region according to a fixed step. Q3 is the result of Q1 and Q2 image processing. The process of image processing is shown in Figure 14, which is called “pixel gray value subtraction” in order to describe it. Every pixel point in the image was processed identically. Each pixel in the resulting image had a gray value of 0, with a gray value of 0 representing black. The final image was all black to visualize the reaction result as correct. The verification results show that picture Q3 is all black, which means that Q1 and Q2 are completely consistent, so the image stitching result is accurate.

**Figure 15:**
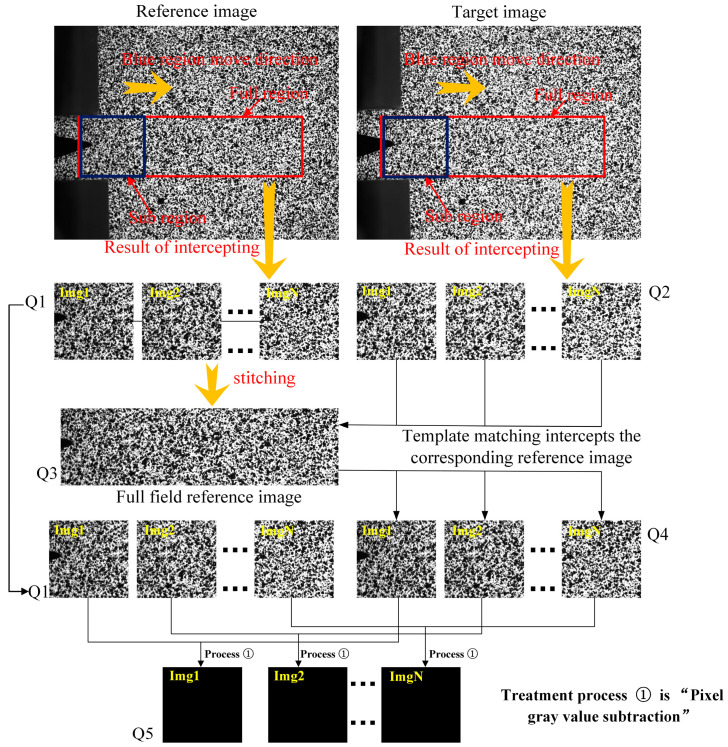
Template matching verification process.

The correctness verification process of template matching is shown in Figure 15. Q1 and Q2 are a set of images truncated according to a fixed step from the red rectangular regions of the reference image and the target image with the crack according to the size of the blue rectangular region, respectively. Q3 is the simulated full-field reference image obtained by stitching the images in Q1. Q4 is a set of corresponding reference images intercepted by template matching in Q3 using each picture in Q2. Every image in Q1 and Q4 is used to calculate the gray subtraction of the corresponding pixel, and the result is Q5. Q5 is a set of all-black images, which indicate that the template matching result is accurate.

### 4.2. In Situ Measurement of the Strain Field at the Fatigue Crack Tip of Q&P980

**Figure 16:**
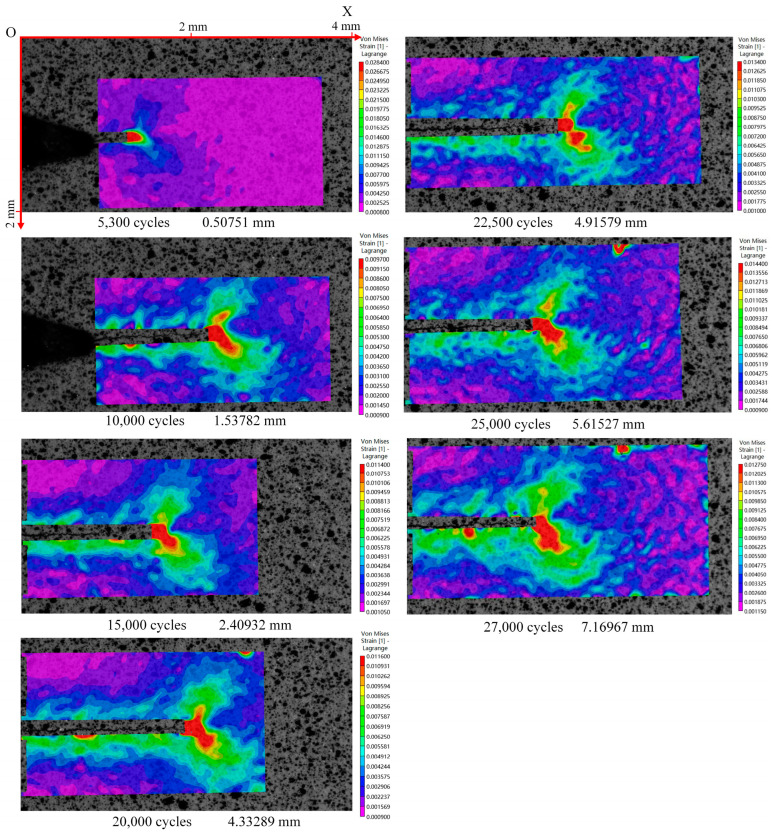
Strain field at maximum load for different numbers of cycles.

The von Mises strain field at the crack tip of seven groups of images at the maximum load was calculated by using the VIC-2D software. The results are shown in Figure 16. The strain field is butterfly-shaped, and the strain concentration is observed at the crack tip. The strain concentration area is skewed, which is shown by the asymmetric butterfly shape of the strain field.

### 4.3. Verification of the Measurement Results of the In Situ Strain Field

**Figure 17:**
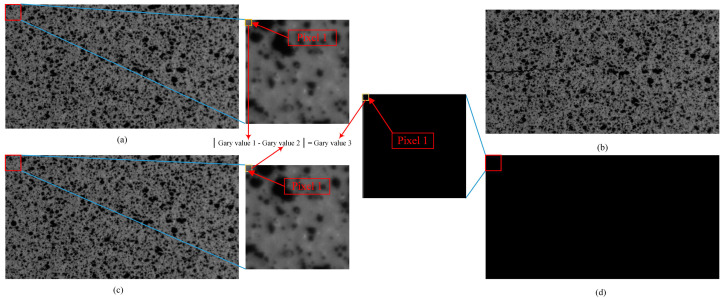
(**a**) Original reference image: (**b**) The captured target image; (**c**) the reference image of template matching; (**d**) the pixel gray value subtraction results of (**a**) and (**c**).

**Figure 18:**
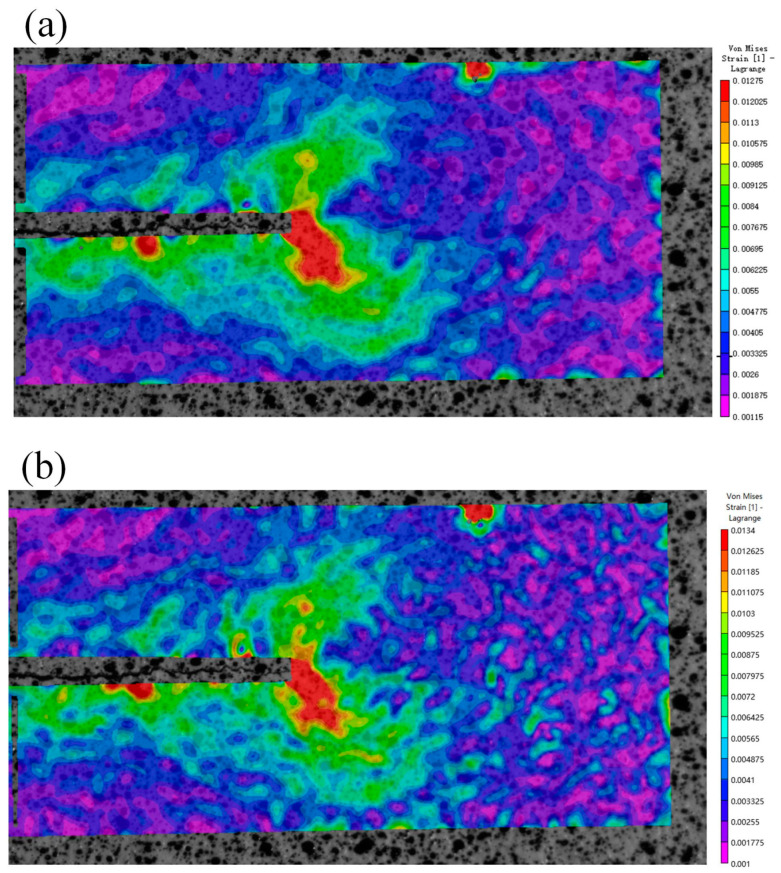
(**a**) The results of the strain field calculated by the template matching reference image; (**b**) the results of the strain field calculated by the reference image of the original acquisition.

Figure 17a is the fourth image in Figure 9, which we call the original reference image. This means that Figure 17a was captured directly by the camera. Figure 17b is the target image at the maximum load in the seventh set of data. Figure 17c is the reference image obtained by template matching with Figure 13 and Figure 17d, which we call the reference images of template matching. This means that Figure 17c was obtained by the template matching algorithm. In order to verify whether Figure 17a,c are consistent, “Pixel gray value subtraction” between Figure 17a,c was carried out. The result in Figure 17d is a black image, showing that Figure 17a,c are exactly the same. Two different reference and target images were used to solve the strain field, and the results are shown in Figure 18. The results show that the shape of the strain field distribution is basically the same.

**Figure 19:**
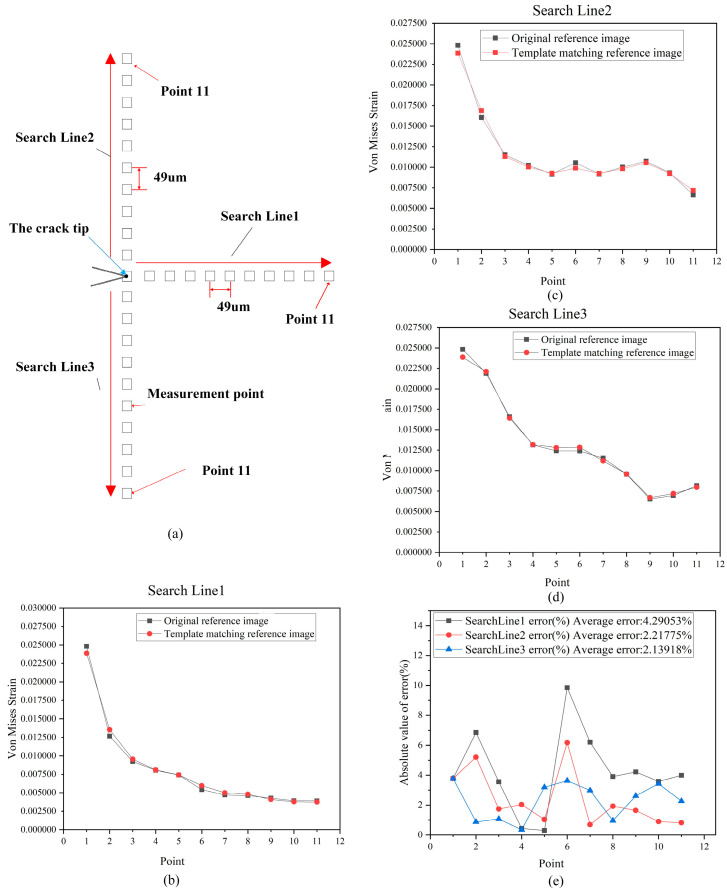
(**a**) Measuring point arrangement; (**b**) strain results of search line 1; (**c**) strain results of search line 2; (**d**) strain results of search line 3; (**e**) measurement error of three search lines.

In order to further analyze the accuracy of the strain field measured by the method proposed in this paper, for the strain field results in Figure 18a,b, the measuring points shown in Figure 19a were arranged at equal intervals. The measuring points with a spacing of 49 μm were arranged directly to the right, above and below the crack tip, and 11 points were arranged on each search line. The numerical results of the strain field solved by different reference images on three search lines were drawn, as shown in Figure 19b–d. The von Mises strain errors measured by the three search lines are shown in Figure 17e. The maximum absolute error did not exceed 10%, and the average error of each search line was 4.3%, 2.2%, and 2.1%, respectively. The numerical consistency of the strain field calculated by DIC from two different reference images is very high. The slight difference is due to the slight deviation caused by the layout of the ROI region when using the DIC strain field calculation software.

### 4.4. Further Analysis and Discussion of the Measurement Results

**Figure 20:**
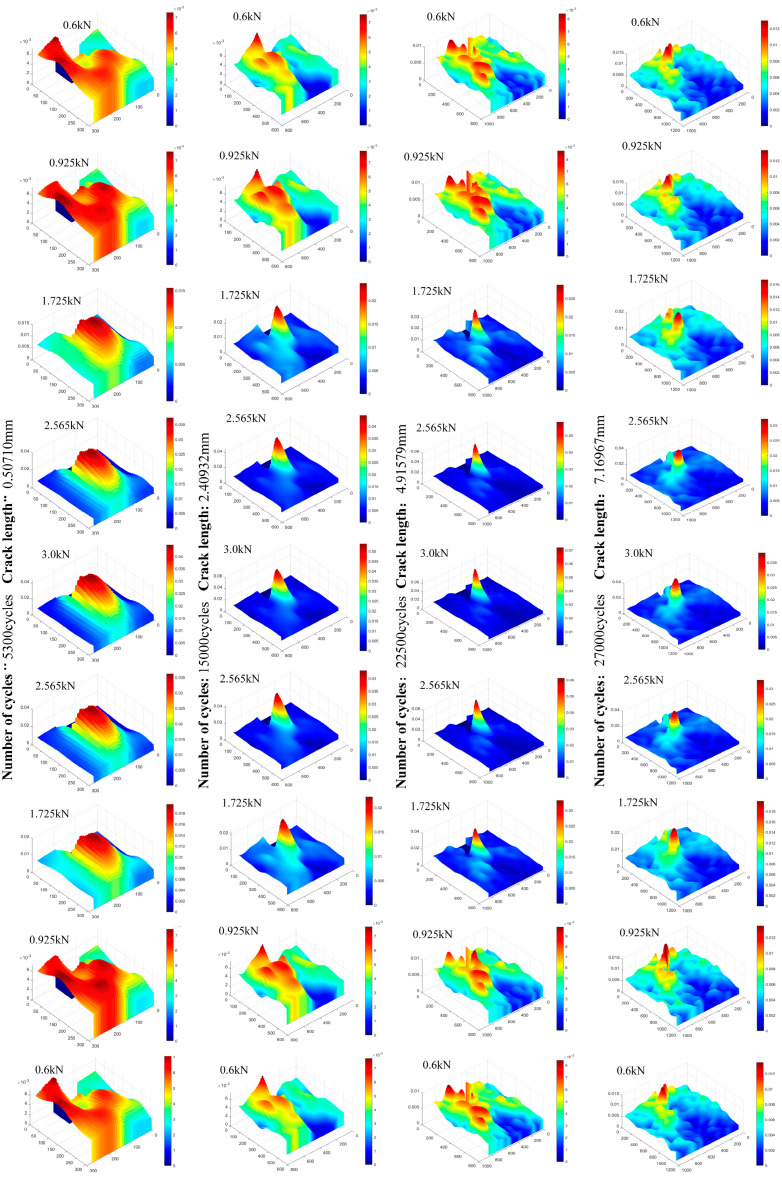
Variation of the von Mises strain field in one period with different crack lengths.

In order to further analyze the evolution law of the strain field, the partial data of the strain field in one load cycle with different crack lengths were plotted in a surface graph. Four sets of von Mises strain data were selected as shown in Figure 20. When the load was small, the strain gradient in the cyclic strain field was small, but there was still obvious strain concentration near the crack tip. With the increase in load, the strain at the crack tip increased sharply and showed a peak on the curved surface, which can be considered as the location of the crack tip. When the strain at the crack tip began to unload after the load peak point, the strain at the crack tip began to decrease, but the decreasing speed was less than the climbing speed under the same load, and the strain at unloading was slightly larger than that at loading. It can be found that the greater the load, the more intense the strain concentration near the crack tip.

After confirming the validity and accuracy of this method, we performed the same test with another material, 316L. In-situ measurement of the strain field was similarly achieved for 316L and 316 stainless steel by the FCG test. The results showed that this method is also suitable for 316L and 316 materials.

## 5. Conclusions

In this paper, based on the combination of the image processing (image stitching and image template matching) and micro-DIC, a method for the in-situ measurement of the microscopic strain field was proposed. This in situ method realizes dynamic tracking measurement of the strain field near the crack tip during crack growth. The feasibility and accuracy of the method were verified by FCG tests. The conclusions are as follows:

(1) Image stitching and template matching technology are accurate, which is embodied the “Original reference image” and “Reference image of template matching” being exactly the same. This result means that the reference images obtained by image stitching and template matching technology are correct.

(2) The average strain error of the three search lines was 4.3%, 2.2%, and 2.1%, respectively, and the maximum error was less than 10%. The results showed that the measured strain field has high accuracy. The in-situ measurement method proposed in this paper is feasible and accurate.

(3) A butterfly shape and strain concentration phenomenon of the strain field near the crack tip were observed. With growth of the crack, the strain concentration became more obvious, and the butterfly wings gradually expanded. For the same crack length, the greater the load, the more obvious the strain concentration. 

(4) The method proposed in this paper does not make higher requirements in regard to the performance of the algorithm used and can be further optimized. In addition, more materials need to be tested, which can improve the usefulness under different conditions.

(5) On the basis of the in-situ strain field measured by this method, the plastic area under the microscopic field of view can be further measured. For example, a method for in situ measurement of the size of the cyclic plastic zone can be studied. In combination with the theoretical analysis, the law of fatigue and fracture of materials can be revealed.

## Figures and Tables

**Table 1:** Material parameters for Q&P980.

Elastic Modulus (E)/GPa	Yield Strength (*σ*)/MPa
197.20	776.48

**Table 2:** Homography matrix for image stitching.

Stitched Image Pair	Homography Matrix
1>>2	$\left[\begin{array}{ccc} 0.9999743338 & 0.0000125326 & 2051.0843781985\\ -0.0000175946 & 0.9999468325 & 0.0000152219\\ -0.0000000751 & 0.0000000427 & 1.0000000000 \end{array}\right]$
2>>3	$\left[\begin{array}{ccc} 1.0000329702 & -0.0000538059 & 2051.0580442528\\ 0.0000112859 & 0.9999752403 & -0.0000118799\\ 0.0000001506 & -0.0000001739 & 1.0000000000 \end{array}\right]$
3>>4	$\left[\begin{array}{ccc} 0.9999580651 & 0.0000120412 & 2051.0991192851\\ 0.0000181374 & 1.0000466528 & -0.0000741406\\ -0.0000000181 & 0.0000003276 & 1.0000000000 \end{array}\right]$

## Data Availability

Not applicable.
